# IPE in LTC Immersion Experience: Creating a Bridge to Careers in Long-Term Care

**DOI:** 10.1093/geroni/igab046.409

**Published:** 2021-12-17

**Authors:** Megan Thomas Hebdon, Christina Wilson, Catherine Bernier-Carney, Jacqueline Telonidis, susan Chase-Cantarini

**Affiliations:** 1 University of Texas at Austin, University of Texas at Austin, Texas, United States; 2 University of Utah College of Nursing, salt lake City, Utah, United States; 3 University of Utah College of Nursing, salt Lake City, Utah, United States; 4 University of Utah College of Nursing, Salt lake City, Utah, United States; 5 University of Utah College of Nursing, Salt Lake City, Utah, United States

## Abstract

The Utah Geriatric Education Consortium seeks to enhance healthcare provider workforce capacity. The purpose of our interprofessional education (IPE) in Long Term Care (LTC) course is to enhance students’ understanding of team-based care for older adults, presenting avenues for career opportunities. The course was offered via distance learning technology to adapt to the COVID-19 pandemic. Health sciences students (n=55) enrolled in the course and completed pre- and post-course work asynchronously. The course also included a two-hour virtual session in partnership with a LTC interprofessional team with a resident case study and discussion using the 4M’s Framework. We will discuss the a) COVID-19 course adaptations, b) IPE case study using the 4M’s Framework to help students conceptualize older adult care, and c) student pre- and post-course discussion responses demonstrating their prior experience with older adults and course impact on their views about caring for older adults in LTC settings.

